# Au/CeO_2_-ZnO/Al_2_O_3_ as Versatile Catalysts for Oxidation Reactions: Application in Gas/Liquid Environmental Processes

**DOI:** 10.3389/fchem.2019.00504

**Published:** 2019-07-12

**Authors:** Cristina Megías-Sayago, Tomas Ramirez Reina, Svetlana Ivanova, Jose A. Odriozola

**Affiliations:** ^1^Departamento de Química Inorgánica, Universidad de Sevilla e Instituto de Ciencia de Materiales de Sevilla, US-CSIC, Sevilla, Spain; ^2^Department of Chemical and Process Engineering, University of Surrey, Guildford, United Kingdom

**Keywords:** gold catalysts, CeO_2_-ZnO/Al_2_O_3_ catalysts, CO oxidation, glucose oxidation, biomass upgrading

## Abstract

The present work showcases the versatility of nanogold systems supported on Zn-doped ceria when applied in two important environmental processes, the total CO oxidation, and the liquid phase oxidation of glucose to gluconic acid. In the CO oxidation the suitability of these materials is clearly demonstrated achieving full conversions even at sub-ambient conditions. Regarding the glucose oxidation our materials display high conversion values (always over 50%) and very importantly full or almost full selectivity toward gluconic acid—an added value platform chemical in the context of biomass upgrading routes. The key factors controlling the successful performance on both reactions are carefully discussed and compared to previous studies in literature. To our knowledge this is one of the very few works in catalysis by gold combining liquid and gas phase reactions and represents a step forward in the flexible behavior of nano gold catalysts.

## Introduction

The strategy to develop efficient gold-based catalysts for a specific reaction involves a careful control of the synthesis parameters as well as the proper support selection to disperse gold nanoparticles. A strong metal-support interaction and the participation of the support in the reaction are also desirable among many other considerations.

The surprising discovery of Haruta et al. (1987) that very small gold nanoparticles exhibit catalytic activity for the oxidation of CO at sub-ambient temperature changed the paradigm on catalysis by gold making it the most used and most popular metal for catalytic CO oxidation reactions (Haruta et al., 1987; Bond and Thompson, 2000; Haruta, 2002; Trim, 2005; Bond et al., 2006; Chen and Goodman, 2006; Cortie et al., 2006; Corti et al., 2007; Corma and Garcia, 2008). Nowadays, a number of possible applications for catalysts able to perform CO oxidation at room temperature (as for example gold based catalysts) appear (Corti et al., 2007). Within the most extended application it should be mentioned: carbon dioxide lasers, gas sensors, respirators for protecting firefighters and miners from the CO poisoning, air-cleaning devices and many other environmentally relevant technologies.

It is well-known that the activity of gold nanoparticles could be enhanced when a high dispersion of gold is achieved due to the improvement of the metal-support interaction and to higher surface/volume ratio, providing more active sites for the catalytic reaction (Centeno et al., 2005). However, the main drawback resides on the kinetic instability of gold nanoparticles and on the high tendency for agglomeration of these nanoparticles at the high temperature required for some applications. Nevertheless, the thermal stability could be improved by a careful choice of the support material, which first role is to disperse and stabilize the nanoparticles against agglomeration (Royer and Duprez, 2011).

The later explains why the choice of the support is of crucial relevance. Generally, gold supported on a reducible transition metal oxide exhibits a significantly enhanced activity in gas phase oxidation reactions compared to gold supported on non-reducible materials due to their ability to supply reactive oxygen (Schubert et al., 2001). In this context, ceria has become one of the most promising supports for oxidation reactions and it has been broadly studied (Carrettin et al., 2004; Jacobs et al., 2004; Lai et al., 2006; Sandoval et al., 2007). The relevance of cerium oxide, as a support for gold nanoparticles, is mainly based on its high oxygen storage capacity resulting from oxygen mobility in its lattice (Trovarelli, 1996). The later is directly correlated to the creation and diffusion of oxygen vacancies, especially on the oxide surface, due to the reversible redox behavior of the Ce^4+^/Ce^3+^couple of CeO_2_. The concentration of these structural defects has been broadly correlated with the catalytic activity in CO elimination reactions (Avgouropoulos and Ioannides, 2008; Xiao et al., 2009; Tabakova et al., 2011). Moreover, these oxygen vacancies may act both as preferential nucleation sites for gold nanoparticles and also as activation points of the molecules, such as CO and O_2_ facilitating the reaction. In some cases these reactive sites can participate in the reaction mechanism by creating activated species from the reactants mixture (e.g., peroxi-like and superoxi-like species) potentiating the oxidation reactions (Wang and Hwang, 2003; Zhang et al., 2009).

In this regard and due to important role of the oxygen vacancies, many authors have proposed some alternatives routes to create them (Tuller and Nowick, 1977; Wuilloud et al., 1984; Panhans and Blumenthal, 1993). Among the different options, for catalytic applications the most popular strategy is to dope ceria with lower valence metallic cations (Gamarra and Martínez-Arias, 2009; Hernández et al., 2010; Laguna et al., 2011; Reina et al., 2013b; Reina, 2014). The formation of oxygen vacancies in a CeO_2_-MO_x_ support is directly related to the formation of Ce-M solid solution. The introduction of dopants in the ceria lattice can provoke structural distortions such as its contraction thus favoring the formation of oxygen vacancies. Certainly, previous studies in our group underlined the importance of these oxygen vacancies and reveal that some cations such as Eu (Hernández et al., 2010), Zn (Laguna et al., 2011; Reina et al., 2012b; Reina, 2014), Fe (Laguna et al., 2011; Reina et al., 2013a,b; Reina, 2014), Cu (Laguna et al., 2012), Zr (Reina, 2014), or Co (Reina et al., 2012a) may favor the formation of these punctual defects leading to promising catalysts in oxidation reactions.

Recently, gold-based catalysts have demonstrated to be promising systems also for the oxidation of organic compounds in liquid media. In this sense, its role in several biomass upgrading routes such as the oxidation of glucose is under investigation. The selective glucose oxidation has been carried out over both unsupported (Comotti et al., 2004; Beltrame et al., 2006) and supported (Biella et al., 2002; Ishida et al., 2008) gold catalysts with good results in terms of activity and selectivity. Lately, some studies have pointed out the support participation during the process (Wan et al., 2014; Megías-Sayago et al., 2016) being most of the studied supports simple or mixed metal oxides such as TiO_2_ (Cao et al., 2015), MgO (Miedziak et al., 2014) Al_2_O_3_, CeO_2_, CeO_2_ (25 wt.%)/ZrO_2_, CeO_2_ (50 wt.%)/ZrO_2_, and CeO_2_ (20 wt.%)/Al_2_O_3_ (Megías-Sayago et al., 2016).

In this work, gold has been deposited over a homemade CeO_2_/Al_2_O_3_ support doped with small amounts of Zn (within the 0–2 wt.% range), targeting a complete Ce-M solid solution formation and generation of oxygen vacancies on the ceria lattice. All the systems have been tested in the total CO oxidation reaction as a quick evaluation of their oxidation skills in gas phase. Afterwards, their activity and selectivity have been evaluated in the selective oxidation of glucose to gluconic acid in liquid media.

## Experimental

### Supports Preparation: *CeO_2_-ZnO/Al_2_O_3_*

The desired amount of Ce(NO_3_)_3_·6H_2_O and Zn precursor Zn(NO_3_)_2_·6H_2_O (Aldrich) were heterogeneously co-impregnated onto γ-alumina powder (Sasol). The co-impregnation was carried out in 50 mL of ethanol, evaporated till obtaining of a dry solid in rotary vapor at reduced pressure and temperature of 50°C. The obtained solid was treated with NH_3_ (Aldrich) solution 10 M during 30 min in order to assure the full conversion of the nitrates to hydroxides precursors. The support was then filtered, dried, and calcined at 500°C for 4 h. The targeted amount of metal oxides supported on alumina was 15 wt.%. Several supports were synthesized in such a way that while keeping constant the 15 wt.% loading the relative proportion of CeO_2_ and ZnO loading changes. ZnO loadings are intended to be in the 0–2 wt.% range. The samples are labeled as CeZnX/Al, where X indicates the theoretical ZnO_x_ loading. For instance, the Au/CeZn_2_/Al solid contains 2 wt.%. Au loading over 15 wt.% of Ce-Zn mixed oxide on Al_2_O_3_ support in which the ZnO_x_ loading is 2 wt.%. Table 1 summarizes the nominal compositions and adopted nomenclatures of prepared supports.

**Table 1:** Nomenclature and nominal composition of prepared supports.

**Sample**	**Nominal values (wt.%)**		
	**CeO**_**2**_	**ZnO**	**Al**_**2**_**O**_**3**_
Al	–	–	100
Ce/Al	15	–	85
Zn/Al	–	15	85
CeZn1/Al	14	1	85
CeZn2/Al	13	2	85

### Gold Deposition

Irrespective of the support the same gold deposition method was utilized in this project, the direct anionic exchange (DAE) method assisted by ammonia (Ivanova et al., 2004). Aqueous solutions of the gold precursor HAuCl_4_ (Johnson Matthey) 2 × 10^−4^ M and the support sieved to 100–200 μm mesh were used in order to obtain a final Au loading of 2 wt.%. The solution was heated to 70°C and aged 20 min. After that, the solution was cooled down to 40°C and 50 mL of NH_3_ (30% Aldrich) were added. The slurry was then filtered, washed with water, dried at 100°C overnight and finally calcined in air at 350°C for 4 h.

In the adopted nomenclature, the real gold loadings are omitted for simplification. The figures' legends account always for the nominal loading values.

### Characterization Techniques

X-ray diffraction (XRD) analysis was performed on an X'Pert Pro PANalytical. Diffraction patterns were recorded with Cu Kα radiation (40 mA, 45 kV) over a 2θ-range of 10–80° and a position-sensitive detector using a step size of 0.05° and a step time of 240 s.

The chemical composition of the samples was evaluated using Panalitycal (AXIOS model) X-ray fluorescence spectrometer equipped with Rh tube of radiation. For the measurements, the samples were dispersed in boric acid pellets.

Temperature-programmed reduction (TPR) experiments were carried out in homemade equipment designed by PID Eng & Tech using a conventional quartz reactor connected to a thermal conductivity detector (TCD). The reactive gas stream, 5% H_2_ in Ar (Air Liquide) was passed through a 50 mg of sample with a flow rate of 50 ml min^−1^ and the temperature rise at 10°C min^−1^ from room temperature to 900°C. A molecular sieve 13X was used to retain the H_2_O produced during the reduction. For quantitative analysis the TCD signal was calibrated with a CuO pattern (Strem Chemicals 99.999%).

High-resolution transmission electron microscopy (HRTEM) and high-angle annular dark field scanning transmission electron microscopy (HAADF–STEM) images were recorded on a JEOL2010F instrument.

### Catalytic Tests

#### Total CO Oxidation (TOX)

The catalytic tests were carried out in a U-shape glass reactor at atmospheric pressure. The catalysts (80 mg) were pre-treated in a 30 mL min^−1^ activation flow of 21% O_2_ balanced in He (from room temperature to 350°C, 5°C min^−1^). After the activation, a reactive flow 3.4% CO (Air Liquide, 99.997%) and 21% O_2_ (Air Liquide, 99.999%) balanced by helium, was passed through the reactor at room temperature. The total gas flow was 42 mL.min^−1^ and the quantitative analysis was carried out with a Blazers OmnistarBentchop mass spectrometer. The catalysts were tested in the reaction flow at room temperature until reached the steady state. Then, the systems were heated to 350°C at 5°C min^−1^.

The same reaction was carried out at temperatures below 0°C. The reactor, loaded with a fresh and activated sample, was immersed into a cooling bath composed by liquid N_2_ and acetone. Once the temperature stabilized, the reactive flow described above was flushed through the reactor. The temperature was increased slowly to room temperature and the quantitative analysis of reactants and products was carried out on a Blazers OmnistarBentchop mass spectrometer calibrated using gas mixtures of CO and CO_2_ in helium. The CO conversion was calculated according to Equation (1) where CO_in_ is the inlet CO concentration and CO_out_ is the one measured at the outlet:

(1) $$\begin{array}{r} Conversion\;\left(\%\right)=\;\frac{CO_{int}-\;CO_{out}}{CO_{int}}\times 100 \end{array}$$

#### Selective Glucose Oxidation

The catalytic tests were carried out in a 50 mL glass batch reactor saturated with oxygen at atmospheric pressure (approximate *P*(O_2_) of 0.1 MPa) with 5 mL 0.2 M glucose solution and Glucose/Au molar ratio of 100. In a typical experiment, a 20 mL/min pure oxygen flux was introduced in the reactor in order to supply an oxygen rich atmosphere. Then, the reactor was closed and the mixture stirred at 600 rpm at 60°C during 18 h without base addition. After reaction, 500 μL of sample was taken from the final mixture, diluted in 500 μL of MilliQ water and immediately analyzed by High-performance liquid chromatography (HPLC). The reported conversions were obtained after comparing the glucose concentration before and after the reaction, (Equation 2). Selectivity was calculated on the base of the carbon moles, as described in Equation (3).

(2) $$\begin{array}{r} Conversion\;\left(\%\right)=\;\frac{{\left[Glucose\right]}_{I}-\;{\left[Glucose\right]}_{F}}{{\left[Glucose\right]}_{I}}\times 100 \end{array}$$

(3) $$\begin{array}{r} Selectivity\;\left(\%\right)=\;\frac{Carbon\;mol\;of\;specific\;product}{Carbon\;mol\;of\;total\;products}\times 100 \end{array}$$

## Results and Discussion

Although the Ce-Zn solid solution is hard to obtain according to the Hume-Rothery rules (William and Hashemi, 2006), it was reported that an epitaxial interaction between both zinc and cerium oxides may create preferential sites for gold deposition in the oxides interface leading to a high gold dispersion and improved catalytic activity (Laguna et al., 2011). The latter suggests a possible promotion of the catalytic activity in presence of ZnO as promoter.

The chemical composition and the specific surface area of the prepared samples are summarized in Table 2. In general, cerium loadings are close to the nominal value (15 wt.%), contrary to the amount of zinc oxide in the doped systems, which resulted to be ~50% of the intended value. The observed ZnO losses could be related to the ammonia treatment employed during the support synthesis which could promote ZnO dissolution. Gold content is close to 2 wt.% except for Au/Al and Au/Zn/Al catalysts for which the gold content is lower than the targeted one. As for the surface area, all samples display comparable values indicating that this parameter is controlled by the primary support, γ-Al_2_O_3_.

**Table 2:** Chemical compositions and specific surface area of the prepared Zn promoted solids.

**Sample**	**CeO**_**2**_ **(wt.%)**	**ZnO** **(wt.%)**	**Al**_**2**_**O_3_** **(wt.%)**	**Au** **(wt.%)**	**Au particle** **size (nm)**	**S**_**BET**_ **(m^2^/g)**
Al	–	–	100		–	202
Au/Al	–	–	98.84	1.16	4	217
Ce/Al	11.1	–	88.91	–	–	186
Au/Ce/Al	10.8	–	87.46	1.7	4	197
Zn/Al	–	2.96	97.04	–	–	184
Au/Zn/Al	–	2.53	96.79	0.69	4	213
CeZn1/Al	15.78	0.47	83.75	–	–	179
Au/CeZn1/Al	13.84	0.40	83.89	1.87	4	192
CeZn2/Al	14.40	1.2	84.40		–	185
Au/CeZn2/Al	13.37	1.05	83.59	1.99	4	181

**Figure 1:**
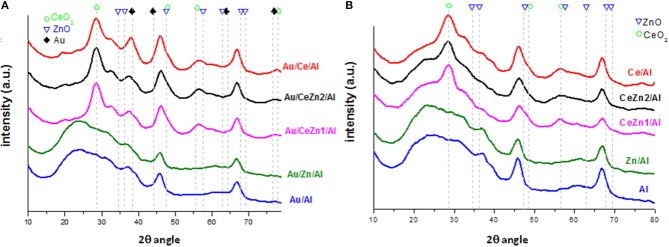
XRD patterns of **(A)** gold catalysts and **(B)** parent supports.

X-ray diffraction (XRD) diffraction patterns of the prepared supports and catalysts are shown in Figure 1. For all systems ceria phase pattern corresponds to the cubic CeO_2_ fluorite type structure (JCPDS# 00-004-0593) and to the γ-Al_2_O_3_ phase (JCPDS# 00-048-0367). Due probably to the small quantity of doping oxide and/or the amorphous character of the ZnO, no signals regarding it were found. Moreover, no formation of solid solution between zinc and cerium oxides was observed. Certainly, Au and Zn introduction did not substantially modify the size of cerium oxide particles which is around 5 nm (Scherrer calculation). Similar diffraction peaks are obtained for gold catalysts (Figure 1B) not being detected any signal related to gold presence. The latter indicates that gold nanoparticles are beyond the detection limit of the equipment, 4 nm, and well-dispersed on the support.

**Figure 2:**
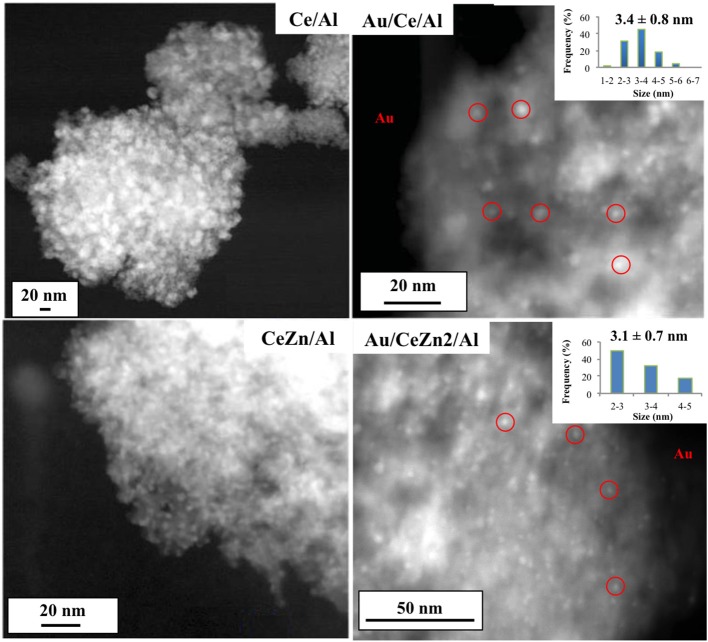
Representative images of supports and catalysts.

Transmission electron microscopy (TEM) images of representative supports and catalysts are presented in Figure 2. White zones observed for supports correspond to heavier elements (Zn, Ce) in comparison with alumina. Due to the lack of contrast, zinc and ceria oxides cannot be distinguished. The same effect is observed when gold is deposited owing to the low mass and diffraction contrast. Nevertheless, gold nanoparticles are recognized and underlined as the white spots (right images in Figure 2). Despite the contrast is not good enough to study and measure the particle size distribution and dispersion, gold size of selected samples was measured over a total of 40 counted particles to confirm the hypothesis (Figure 2, inset). As can be seen, particles sizes are 3.4 ± 0.8 nm and 3.1 ± 0.7 nm for Au/CeAl and Au/Ce/Zn2/Al, respectively, in good agreement with XRD results. Taking into account the standard deviation and considering the difficulty to measure gold nanoparticles due to the lack of contrast, the particle size is assumed to be 4 nm in all samples as shown in Table 2.

**Figure 3:**
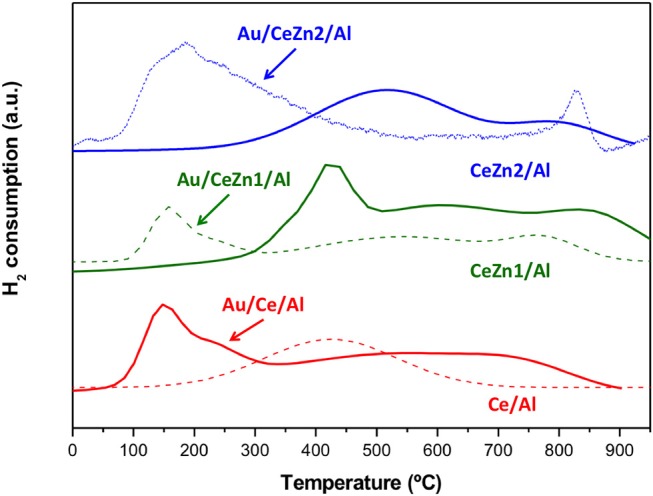
TPR-H_2_ profiles of the supports (full lines) and their corresponding gold catalysts (dashed lines).

An approach to understand the redox properties of the prepared systems was obtained by means of hydrogen temperature-programmed reduction (H_2_-TPR, Figure 3). Al and Zn/Al samples did not exhibit any reduction process. Only ceria containing materials presented a TPR profile. Ce/Al system shows only one broad reducibility zone between 200–600°C ascribed to the reduction of the surface ceria. The addition of Zn increases ceria reducibility accounting for the second reduction zone at higher temperatures in the CeZn/Al samples. In other words, when Zn is present the reduction of ceria bulk (which correspond to the higher temperatures process) occurs confirming the enhanced reducibility of the Zn doped samples. Regarding the gold catalysts, as expected the addition of gold shifts the reduction zones toward lower temperatures. Gold nanoparticles facilitates ceria reduction. For all the catalysts, both reduction processes (surface and bulk) are manifested.

The quantification of the total reduction was made using Equation (4). The Reduction Percentage (RP) relates the experimentally measured H_2_ consumption (E_HC_) to the total theoretical H_2_ consumption (T_HC_).

(4) $$\begin{array}{r} RP\;\left(\%\right)=\;\frac{E_{HC}}{T_{HC}}\;x\;100 \end{array}$$

**Figure 4:**
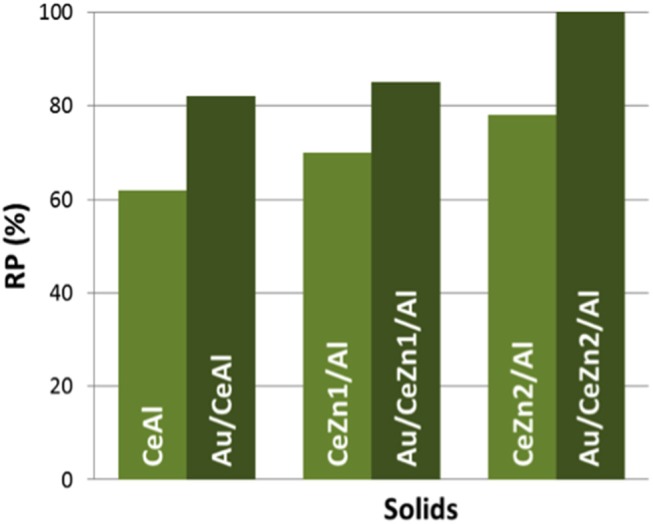
Reducibility percentage (RP) of the prepared solids.

Reduction Percentage (RP) values of the prepared materials are presented in Figure 4. For the calculation, as no reduction of ZnO was observed, only ceria reduction is considered i.e., Ce^4+^ to Ce^3+^. When gold is present in the samples, its reduction is discarded, and considered that its full reduction from Au^3+^ to Au^0^ occurs during the calcination of the samples. Among the supports the total RP grows with the inclusion of ZnO. The CeZn2/Al sample is the one presenting the highest reducibility. Definitely ZnO promotes CeO_2_ redox properties. The boosting effect of gold in the reducibility is also evidenced in Figure 4. All the gold catalysts presented higher RP values than their parent supports. In the case of the Au/CeZn2/Al complete reduction was achieved. It can be concluded that this sample presents the best redox properties within the prepared series.

**Figure 5:**
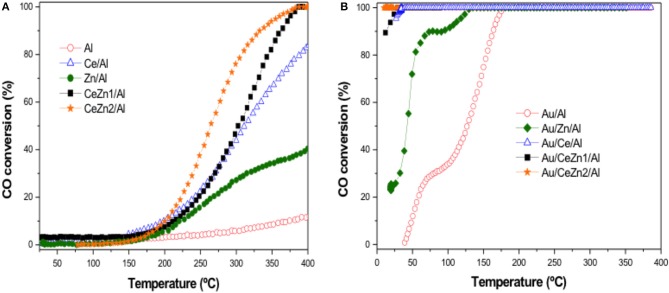
CO oxidation activity **(A)** supports test **(B)** gold catalysts.

The catalytic screening in the total oxidation of CO of the studied solids is presented in Figure 5. For the supports, the addition of ZnO to the bare Al_2_O_3_ increased the activity in the oxidation process. Despite no reducibility was observed for the Zn/Al solid, it seems that ZnO can participate in the CO oxidation process. Indeed the formation of oxygen vacancies in the ZnO phase was proposed by Laguna et al. (2011) as follows:

$$\begin{array}{l} \text{ZnO }⇌\text{ Zn}{\text{O}}_{1-\text{x}}+{{\text{V}}_{\text{o}}}^{\text{x}}+\text{x}/2{\text{ O}}_{2} \end{array}$$

where ${\text{V}}_{\text{o}}^{\text{x}}$ is an oxygen vacancy on the ZnO lattice. These punctual defects may act as O_2_ and CO activation sites. Furthermore, this process involves an additional oxygen contribution coming from ZnO lattice that can participate and benefiting the CO oxidation process. The activity of the supports is clearly superior when ceria is present. The presence of cerium oxide is mandatory for a good performance in the catalytic process. Here the promotional effect of ZnO to CeO_2_ is manifested. Ce-Zn mixed systems supported on alumina are considerably more active than the Ce/Al solid. The activity data totally agrees with the reducibility features of the presented systems, i.e., the higher the reducibility, the better the CO oxidation performance.

The activity of the gold supported catalysts is shown in Figure 5B. All the ceria containing samples reached total CO conversion at room temperature revealing their high capacity for CO abatement. In contrasts, Au/Zn/Al and Au/Al presented poorer activity than the ceria containing systems being Au/Zn/Al better than the Au/Al sample.

**Figure 6:**
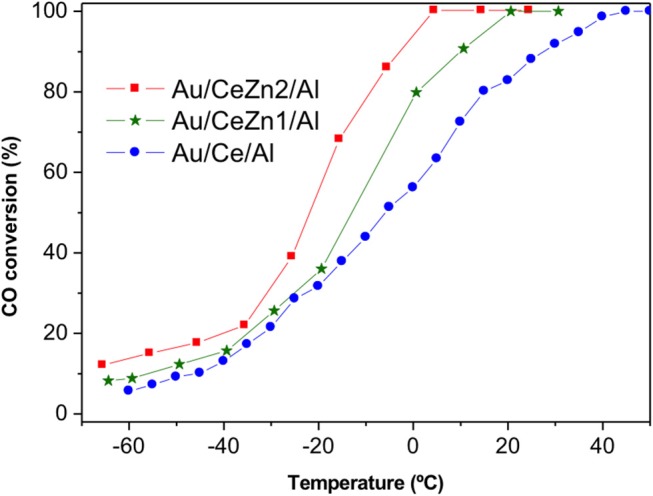
Sub-ambient CO oxidation test.

In order to discriminate among the ceria containing samples, a sub-ambient CO oxidation test was carried out and the results are presented in Figure 6. All the catalysts are very efficient in the CO elimination reaching complete CO conversion at 2°C in the best situation. Here again, the ZnO to ceria promotion is evidenced. The catalytic activity follows the next order:

$$\begin{array}{l} \text{Au/CeZn2/Al }›\text{Au/CeZn1/Al }›\text{ Au/Ce/Al} \end{array}$$

The boosting effect of ZnO in the CO oxidation is related to the improved reducibility exhibited for the Zn-doped samples respect to the un-doped Au/Ce/Al. Once again, this result highlights the importance of the catalysts' redox skills features for achieving a good catalytic behavior in the CO elimination reactions.

Interesting information is extracted when Figure 5 and Figure 5B are compared. If the attention is focused on the shape of the curves, it can be observed that ceria containing samples present a single sigmoidal-like curve while Au/ZnO/Al and Au/Al presented a double sigmoidal plot. This fact is related to the mechanistic aspects behind the CO oxidation process and it is worth to briefly comment it. The CO oxidation mechanism has been broadly addressed but may be one of the best summaries can be found in Bond and Thompson's (2009). Herein, two main reaction mechanisms are proposed: (1) The CO oxidation over Au supported on non-reducible materials as for example Au/Al_2_O_3_ and (2) the Mars-Van Krevelen mechanisms which control the reaction when gold is supported on reducible materials like ceria. Two important differences must be noted: (1) In the first situation the support does not participate in the reaction and it is only used as a physical matrix to disperse gold nanoparticles. However, when the support is reducible, its oxygen vacancies play a determinant role in the oxygen activation; (2) In the first mechanism water is needed to complete the catalytic cycle while the presence of water is not necessary when gold is supported on reducible materials. This second feature accounts for the differences showcase in the CO oxidation curves. Examining carefully the CO oxidation curves of Au/Al and Au/Zn/Al one can realize that the second sigmoidal process starts at temperatures close to 100°C. This is due to the participation of water coming from the sample or formed from the hydroxyl groups of the support. On the other hand, the single sigmoidal shape of the ceria containing materials supports that the reaction most probably proceed under Mars-Van Krevelen pathways with the participation of the support.

**Scheme 1:**
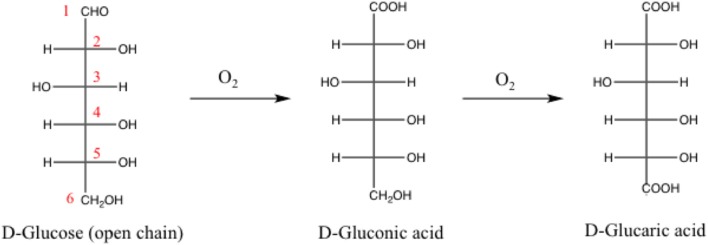
Consecutive oxidation of glucose to gluconic and glucaric acids (Fischer projections).

Sure enough our multicomponent catalysts are very effective materials for a relevant gas phase oxidation processes such as CO oxidation. Yet more if carefully designed (i.e., controlling gold particle size and enhancing the redox properties) excellent activity can be attained even at sub-ambient conditions. At this point some questions arise, would these catalysts be expected to have the same behavior in liquid-phase media? Are they versatile materials further applicability in other environmental processes? Aiming to tackle these queries, the catalytic viability of the ZnO doped systems was evaluated in the selective oxidation of glucose at atmospheric pressure. Such reaction is considered one of the most studied routes to efficiently transform lignocellulosic derivatives into platform chemicals. In general, the use of vegetal biomass to produce value added chemicals is widely recognized as a potential alternative of the petrochemical routes—attracting the attention of the scientific community in the last two decades (Climent et al., 2011, 2014; Lanzafame et al., 2012). The production of gluconic acid platform molecule from glucose (Scheme 1) entails the selective oxidation of the aldehydic function at the anomeric position (C1), remaining unaltered all the alcoholic groups (C2–C6) present in the molecule. Derived from its multifunctionality, the choice of a robust and highly selective catalyst is imperative in order to avoid side products (Wojcieszak et al., 2016). The most common byproduct is glucaric acid, presented also in Scheme 1.

The catalytic results obtained after 18 h at 60°C are summarized in Table 3. In order to compare them properly and due to the slightly different gold loading within the studied series, the glucose conversion is normalized per moles of gold. As general observation, the catalysts exhibit a different behavior when tested in aqueous media. Alcohol oxidation follows a very different trend most probably due to a different oxidation mechanism. It should be underlined that all systems are very selective toward gluconic acid formation, with exception of Au/Al sample for which 91% selectivity is observed. The observed results in terms of glucose converted per Au mole showcase the following activity trend:

$$\begin{array}{l} \text{Au}/\text{Zn}/\text{Al}>\text{Au}/\text{CeZn}1/\text{Al}>\text{Au}/\text{CeZn}2/\text{Al}\approx \text{Au}/\text{CeAl}\approx \text{Au}/\text{Al} \end{array}$$

**Table 3:** Conversion and selectivity values obtained over Au/CeO_2_-ZnO/Al_2_O_3_ materials.

**Catalyst**	**Conversion (%)**	**Glucose converted/molesAu**	**Selectivity (%)**	
			**Gluconic acid**	**Glucaric acid**
AuAl	58	69	91^*^	0
Au/CeAl	80	68	99	1
Au/Zn/Al	56	160	100	0
Au/CeZn1/Al	74	78	100	0
Au/CeZn2/Al	70	69	100	0

* *9% lactic acid selectivity*.

The highest performance obtained for Au/Zn/Al could be attributed to both the lower gold uptake and the highest Zn content. The former would give an idea about the optimum gold loading for the amount of used reactant while the latter exposes the importance of support Lewis acidity in this process. In this regard, the studies carried out by Gutiérrez-ortiz et al. (2004) demonstrated that the oxidation activity of C_2_ chlorohydrocarbons over ceria-zirconia catalysts is function of the Ce/Zr ratio. The incorporation of higher amounts of zirconium into ceria lattice shows complex influence on acidity of the pure parent oxide. Pure ceria presents the lowest acidity, markedly increased upon zirconia addition and the sample the highest concentration of incorporated zirconia presents the strongest Lewis acidity. Indeed, Corma et al. (Abad et al., 2005) further confirmed that the first alcohol oxidation step starts when the alcohol reacts with solid's Lewis acid sites yielding a metal alkoxide. All these findings help us to explain why Zn containing samples exhibit a better performance, which does not seem to be related with the oxygen mobility.

## Conclusions

This work evidences the versatility of multicomponent Au-CeO_2_ based catalysts for environmental catalysis applications. The results obtained here validate the use of small amounts of ZnO as a ceria promoter. Despite no Ce-Zn solid solution was achieved an intimate Ce-Zn contact as a consequence of the homogeneity of the samples is obtained. The samples present small and homogenously distributed gold nanoparticles on the ZnO-promoted ceria support. The results presented in the TPR analysis show the boosted reducibility of the Zn containing samples compared to the un-promoted one. These skills result in very promising systems for CO oxidation reaching complete conversions of CO even at sub-ambient temperatures. A linear correlation between reducibility and CO oxidation activity was found in such a way that higher the reducibility, better the CO oxidation performance. All the ceria containing samples are much more efficient that the Au/Al and Au/Zn/Al ones. The role played by the support has been manifested. The shape of the obtained curves points different reaction mechanisms within the series.

The catalysts were successfully tested in liquid-phase, i.e., in the glucose oxidation reaction in aqueous media showcasing very promising results in terms of activity and selectivity toward gluconic acid—a highly important platform chemical in the context of modern bio-refineries. In contrast to the CO oxidation process, the activity for glucose oxidation seems to be more influenced by the acid-base properties rather than the redox capabilities of the materials—an interesting observation to be considered for further improvement of the catalysts design.

The power of gold based catalysts is evidenced once again in this work whose overriding idea is to demonstrate the flexibility of nano gold for environmental catalysis. The suitable support choice and the present of effective promoters such as ZnO result in highly efficient catalysts for both gas and liquid phase reactions with potential application in gas processing and biomass upgrading technologies.

## Data Availability

All datasets generated for this study are included in the manuscript/supplementary files.

## Author Contributions

All authors listed have made a substantial, direct and intellectual contribution to the work, and approved it for publication.

### Conflict of Interest Statement

The authors declare that the research was conducted in the absence of any commercial or financial relationships that could be construed as a potential conflict of interest.
